# Long-term outcomes of frontline imatinib therapy for chronic myeloid leukemia in China

**DOI:** 10.3389/fonc.2023.1172910

**Published:** 2023-05-02

**Authors:** Fang Cheng, Guolin Yuan, Qiang Li, Zheng Cui, Weiming Li

**Affiliations:** ^1^ Department of Pharmacy, Union Hospital, Tongji Medical College, Huazhong University of Science and Technology, Wuhan, China; ^2^ Hubei Province Clinical Research Center for Precision Medicine for Critical Illness, Wuhan, China; ^3^ Hubei Key Laboratory of Biological Targeted Therapy, Union Hospital, Tongji Medical College, Huazhong University of Science and Technology, Wuhan, China; ^4^ Department of Hematology, Xiangyang Central Hospital, Affiliated Hospital of Hubei University of Arts and Science, Hubei, China; ^5^ Department of Hematology, Union Hospital, Tongji Medical College, Huazhong University of Science and Technology, Wuhan, China

**Keywords:** imatinib, chromic myeloid leukemia, pharmacotherapy, efficacy, drug safety

## Abstract

**Background:**

Imatinib is the first-line therapy recommended for chronic myeloid leukemia (CML) patients in China. We reported a long-term follow-up study of patients on imatinib as first-line treatment for chronic phase (CP) CML to provide an important reference for the actual clinical treatment regimen of CML patients in China.

**Methods:**

We evaluated the long-term efficacy, safety, low-dose attempt after years of treatment, and treatment-free remission (TFR) of 237 CML-CP patients receiving first-line imatinib therapy.

**Results:**

The median age was 46 years (interquartile range: 33–55). After a median follow-up of 6.5 years, the cumulative complete cytogenetic response, major molecular response (MMR), and MR4.5 rates were 82.6%, 80.4%, and 69.3%, respectively. The 10-year transformation-free, event-free, and failure-free survival rates were 97.3%, 87.2% and 53.5%, respectively. Fifty-two (21.9%) patients with sustained deep molecular response (DMR) were treated with low-dose imatinib after years of imatinib treatment. No significant differences in the 1-year and 2-year molecular relapse-free survival in MMR and MR4 were observed between the standard-dose and low-dose groups. Twenty-eight (11.8%) patients discontinued imatinib, and the median time to maintain DMR before discontinuation was 8.43 years. Thirteen patients (5.5%) remained in TFR for a median of 43.33 months. No patients transformed to accelerate or blast phase or died. No new, late toxicity was observed, and the most frequent grade 3/4 adverse events were neutropenia (9.3%), anemia (7.6%), thrombocytopenia (6.3%), and rash (4.2%).

**Conclusion:**

This study confirmed the long-term efficacy and safety of imatinib for treating Chinese CML patients. Additionally, it demonstrated the feasibility of imatinib dose reduction and TFR attempts in patients with sustained stable DMR after years of imatinib treatment in real-life settings.

## Introduction

Chronic myeloid leukemia (CML) is a hematologic malignancy that originates from a clonal proliferation of bone marrow hematopoietic stem cells, characterized by an oncogenic Philadelphia (Ph) chromosome carrying the BCR-ABL1 fusion gene (1). Imatinib mesylate, a first-generation tyrosine kinase inhibitor (TKI), was approved by the US Food and Drug Administration in 2001 and has changed the landscape of CML treatment, significantly improving long-term prognosis and overall survival (OS) of patients with CML (2).

Second-generation TKIs are mainly designed to overcome imatinib resistance, and they lead to more rapid and profound molecular responses at early time points than imatinib (3, 4). However, whether the second-generation TKIs can induce better long-term clinical outcomes remains unclear. Currently, the TKIs approved in China for treating CML mainly include imatinib, dasatinib, nilotinib, flumatinib, and olverembatinib. When evaluating the treatment options for newly diagnosed chronic phase (CP) CML patients, clinical prognoses such as progression-free survival (PFS) and OS, safety, cost, patient’s treatment goal, and possible discontinuation must be comprehensively considered. Imatinib is the recommended first-line treatment for CML patients in China. To better understand the spectrum of imatinib use in Chinese CML patients, we report on a long-term follow-up study on imatinib as the first-line treatment for CML-CP. We aimed to provide an important reference for the actual clinical treatment regimen of CML patients in China.

## Materials and methods

### Patients

In total, 237 CML patients who received first-line imatinib treatment from January 2011 to December 2021 were enrolled. The inclusion criteria included age over 18 years old, receiving imatinib as first-line therapy, and good compliance. Good compliance was defined as taking imatinib on time and in the proper amount every day, and attending follow-up visits regularly. Pharmacists conducted compliance surveys every 3-6 months, and performed medication education for patients with poor compliance. Patients with initial diagnoses of accelerated phase (AP) and blast phase (BP), first-line treatment with second-generation TKIs, or incomplete data were excluded. The patients’ basic information, laboratory findings, medication, adverse reactions, and other clinical data were obtained during regular follow-ups at an outpatient clinic.

### Drug administration

Imatinib was administered orally at a fixed time, starting at 400 mg once daily. Low-dose therapy was selected to prevent adverse events (AEs), reduce financial burden, or prepare before discontinuation in patients with sustained optimal response. Patients with sustained deep molecular response (DMR) for more than two years could consider discontinuing imatinib under the guidance of physicians. Patients with intolerable AEs, suboptimal response, or treatment failure could switch to second or third-generation TKIs (dasatinib, nilotinib, flumatinib, or olverembatinib).

### Clinical response assessment

CML-CP was defined as less than 10% blasts in the peripheral blood or bone marrow, and the absence of extramedullary involvement. *BCR-ABL1* mRNA levels can be detected by cytogenetic and molecular tests to evaluate the patient’s clinical response to imatinib treatment. Optimal response, suboptimal response, and treatment failure were evaluated based on the cytogenetic and molecular response at 3, 6, and 12 months after imatinib treatment, and categorized into three groups as per ELN 2020 guidelines (5). Optimal MR included BCR-ABL ≤10%, ≤1%, and ≤0.1% at 3, 6, and ≥12 months, respectively. A warning was defined as BCR-ABL >10%, >1% to 10%, and >0.1% to 1% at 3, 6, and ≥12 months, respectively. Failure was defined as BCR-ABL >10% at 3 months, if confirmed within 1–3 months, BCR-ABL >10% at 6 months, and BCR-ABL >1% at 12 months and anytime. Complete cytogenetic response (CCyR) indicates that 0% Ph^+^ metaphases in the bone marrow, and 0%–35% Ph^+^ metaphases is attributed to major cytogenetic response (MCyR). Major molecular response (MMR) was defined as *BCR-ABL1* ^IS^ ≤0.1%. DMR included either MR4.0 (*BCR-ABL1* ^IS^ ≤0.01%) or MR4.5 (*BCR-ABL1* ^IS^ ≤0.0032%) or undetectable BCR-ABL1 transcripts. Molecular relapse-free survival (MRFS) in MMR and MR4 indicated the probability of survival in patients with sustained MMR and MR4, respectively. Molecular recurrence was defined as loss of MMR at any time. In addition, transformation-free survival (TFS) was defined as transformation to AP and BP or death during treatment. Furthermore, event-free survival (EFS) was defined as the time from the initiation of imatinib therapy to any of the following events: loss of complete hematological remission (CHR), loss of MCyR or CCyR, loss of MMR, exhibition of mutations, clonal chromosome abnormalities in Ph^+^ cells, progression to AP or BP, or death for any cause at any time. Moreover, failure-free survival (FFS) was defined as the time from initiating imatinib therapy to EFS-defining events, including a lack of CCyR at 18 months, lack of MCyR at 12 months, or discontinuation therapy for any reason, including switching to different TKIs. Finally, treatment-free remission (TFR) was defined as the duration between discontinuing imatinib and restarting TKIs.

### Adverse reactions

Patients were followed up regularly for hematological and biochemical examinations. We documented AEs that occurred after taking imatinib, such as hematological toxicities (leukopenia or neutropenia, thrombocytopenia, and anemia), gastrointestinal symptoms (nausea, vomiting, diarrhea, and abdominal pain), periorbital and limb edema, rash, fatigue, musculoskeletal pain, conjunctival hemorrhage, metabolic syndrome (hyperglycemia, dyslipidemia, and hyperuricemia), hepatobiliary and kidney dysfunction, and skin whitening. The severity of AEs was graded according to the Common Terminology Criteria for Adverse Events, version 4.0.

### Statistical analysis

Categorical variables were presented as frequencies and percentages, and continuous data were described using the median and interquartile range (IQR). The Kaplan–Meier method and log-rank tests were used to estimate survival probabilities and compare MRFS between the standard-dose and low-dose groups. IBM SPSS Statistics, version 25.0 (IBM Corp., Armonk, N.Y., USA) software was used for statistical analyses, and p-value <0.05 was considered significant.

## Results

### Patient characteristics

Demographic and clinical information are shown in **Table 1**. Among the 237 patients, 121 (51.1%) were males. The median age was 46 years (IQR: 33–55). The median duration of imatinib therapy was 6.28 years (IQR: 3.96–9.98). Hypertension (N=14 [5.9%]) and diabetes (N=11 [4.6%]) were the most common pre-existing diseases. A total of 105 (44.3%) patients received imported brand imatinib, and 126 (53.2%) patients received domestic imatinib. Fifty-two (21.9%) patients were treated with low-dose imatinib, 87 (36.7%) patients switched to second or third-generation TKIs, 28 (11.8%) patients discontinued imatinib, and one (0.1%) patient died from other cause.

**Table 1:** Demographic and clinical characteristics of patients with chronic myeloid leukemia.

Variables	
Sex (male), n (%)	121 (51.1)
Age, years, median (IQR)	46 (33–55)
Duration of imatinib therapy, year, median (IQR)	6.3 (4.0–10.0)
Follow-up, year, median (IQR)	6.5 (4.2–10.3)
Comorbidity, n (%)	
Hypertension	14 (5.9)
Diabetes	11 (4.6)
Coronary heart disease	8 (3.4)
Hepatitis B	9 (3.8)
Hyperuricemia	7 (3.0)
Others	16 (6.8)
**First-line imatinib**	
imported brand	105 (44.3)
domestic generic	126 (53.2)
Veenat (India)	6 (2.5)
Time to molecular response, month, median (IQR)	
CCyR	7.1 (3.0–7.1)
MMR	11.6 (6.3–15.8)
MR4	18.7 (7.2–30.4)
MR4.5	33.7 (18.5–60.7)
Current status	
Full-dose imatinib therapy	69 (29.1)
Low-dose imatinib therapy^a^	52 (22.0)
Imatinib discontinuation^b^	28 (11.8)
Switch to second-generation TKI^c^	87 (36.7)
Death	1 (0.4)

^a^Low-dose imatinib was defined as 300 mg/day or 200 mg/day.

^b^A total of 28 patients attempted imatinib discontinuation therapy, and 15 patients resumed TKIs treatment.

^c^Four of the patients who switched to second-generation TKIs progressed to the accelerated phase.

CML, chronic myeloid leukemia; IQR, interquartile range; TKI, tyrosine kinase inhibitor; CCyR, complete cytogenetic response; MMR, major molecular response; MR, molecular response.

### Efficacy

Analyses of clinical responses over time are shown in **Table 2**. The response rates at 3, 6, and 12 months after starting therapy were 40.5%, 56.5%, and 62.4%, respectively. During a median follow-up of 6.5 years, the cumulative CCyR, MMR, and MR4.5 rates were 82.6%, 80.4%, and 69.3%, respectively. The median time to CCyR, MMR, MR4, and MR4.5 or better were 7.05 months (IQR: 3.00-7.13), 11.6 months (IQR: 6.34-15.76), 18.69 months (IQR: 7.23-30.35), and 33.7 months (IQR: 18.50-60.70 months), respectively.

**Table 2:** Clinical responses to imatinib over time.

		No. of Patients (%)			
Follow-Up Duration	Evaluable Patients	CCyR	MMR	MR4	MR4.5
3 month	237	96 (40.5)	19 (8.0)	0	0
6 months	237	134 (56.5)	54 (22.8)	29 (12.2)	3 (1.3)
1 year	206	148 (71.8)	110 (53.4)	52 (25.2)	22 (10.7)
2 years	185	160 (86.4)	128 (69.2)	104 (56.2)	58 (31.4)
4 years	156	129 (82.7)	116 (74.4)	100 (64.1)	79 (50.6)
6 years	108	96 (88.9)	88 (81.5)	80 (74.1)	69 (63.9)
8 years	84	75 (89.3)	71 (84.5)	66 (78.6)	54 (64.3)
10 years	55	50 (90.9)	48 (87.3)	43 (78.2)	38 (69.1)

CCyR, complete cytogenetic response; MMR, major molecular response; MR, molecular response.

Among the 87 (36.7%) patients who changed to second or third-generation TKIs, 29 (33.3%) had unfavorable responses, 20 (23.0%) had intolerable adverse reactions, 14 (16.1%) with loss their optimal response during imatinib treatment, five (5.7%) aimed to pursue TFR, 13 (14.9%) developed ABL-kinase domain mutation, four (4.6%) progressed to AP, and two (2.3%) restarted treatment after imatinib discontinuation. In addition, 15 (17.2%), 19 (21.8%), and 43 (49.4%) patients reached CCyR, MMR, and DMR. However, seven (8.0%) entered clinical trials of new drugs after receiving various TKIs treatment (**Table 3**).

**Table 3:** Characteristics of patients who switch to other TKIs.

Reasons for switching to other TKIs, N = 87 (36.7%)	No. of Patients (%)
**Toxicity**	20 (23.0)
Myelosuppression	9 (10.3)
Diarrhea	2 (2.3)
Edema	2 (2.3)
Headache	1 (1.1)
Rash	5 (5.7)
Renal insufficiency	1 (1.1)
**Restarted treatment after relapse**	2 (2.3)
**Pursue TFR**	5 (5.7)
**Loss of response**	31 (35.6)
ABL-kinase domain mutation	13 (14.9)
Loss of MMR	12 (13.8)
Loss of DMR	2 (2.3)
Progression to AP	4 (4.6)
**Poor efficacy**	29 (33.3)
Suboptimal response	17 (19.5)
Failure	12 (13.8)
Subsequent therapy^a^	
Dasatinib	45 (51.7)
Nilotinib	28 (32.2)
Flumatinib	36 (41.4)
Olverembatinib	2 (2.3)
TKIs Treatment line	
2nd line	63 (72.4)
3rd line	19 (21.8)
Successive lines	5 (5.8)
Current status	
CCyR	15 (17.2)
MMR	19 (21.8)
DMR	43 (49.4)
Entering clinical trials of new drugs	7 (8.0)
Lost to follow-up	3 (3.4)

^a^Some patients received various TKIs treatment during the subsequent therapy.

MR, molecular response; CCyR, complete cytogenetic response; MMR, major molecular response; DMR, deep molecular response; AP, accelerated phase; TFR, treatment-free remission.

**Figure 1:**
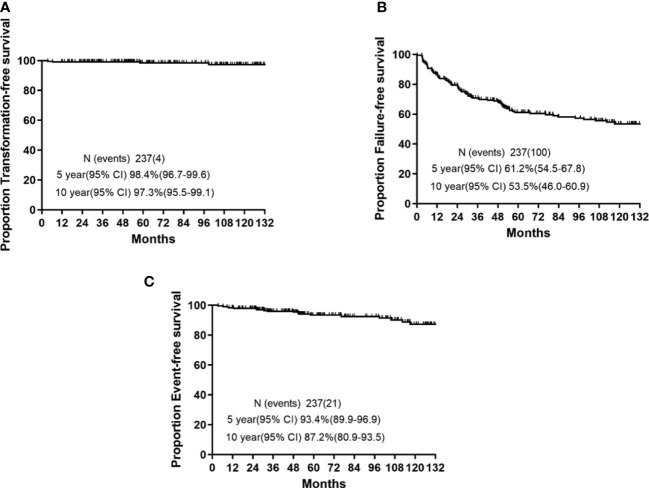
5-year and 10-year survival rates in chronic myeloid leukemia patients who received imatinib as initial therapy. **(A)** Transformation-free survival (TFS). **(B)** Event-free survival (EFS). **(C)** Failure-free survival (FFS).

The 5-year survival probabilities are illustrated in **Figure 1**. The 5-year TFS, EFS, and FFS were 98.4% (95% confidence interval (CI): 96.7–99.6), 93.4 (95% CI: 89.9–96.9), and 61.2% (95% CI: 54.5–67.8), respectively. Moreover, the 10-year TFS, EFS, and FFS were 97.3% (95% CI: 95.5–99.1), 87.2 (95% CI: 80.9–93.5), and 53.5% (95% CI: 46.0–60.9), respectively.

### Low-dose imatinib therapy

**Figure 2:**
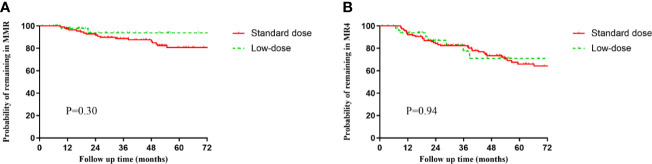
Molecular relapse-free survival (MRFS) of major molecular response (MMR) (*BCR-ABL1* ^IS^ ≤0.1%) or MR4 (*BCR-ABL1* ^IS^ ≤0.01%) in the standard-dose and low-dose groups. **(A)** MMR. **(B)** MR4.

A total of 52 (21.9%) patients received low-dose imatinib therapy (**Table 4**). Thirteen and 39 patients received reduced doses of 300 and 200 mg/day, respectively. Eight (15.3%) patients with sustained MR4 and 44 (84.6%) with sustained MR4.5 or better were on initiated low-dose therapy. The duration of imatinib therapy before dose reduction was 6.95 ± 3.44 years. The main reasons for dose reduction were toxicity (80.7%) associated with imatinib, preparation before discontinuation (7.7%), and financial reasons (11.5%). Two and three patients experienced a loss of MMR and DMR during low-dose treatment, respectively. No significant differences in the 1-year and 2-year MRFS in MMR (**Figure 2A**) and MR4 (**Figure 2B**) were observed in the low-dose compared with the standard-dose group (1 year MMR—97.8% *vs* 96.4%, 2 years MMR—93.7% *vs* 91.7%; 1 year MR4—93.8% *vs* 92.2%, 2 years MR4—87.1% *vs* 84.2%). Importantly, among the 42 patients who received reduced doses due to AEs, 36 (85.7%) reported that their AEs resolved or were significantly improved after dose reduction.

**Table 4:** Low-dose imatinib therapy.

Low-dose imatinib therapy, N = 52 (21.9%)	No. of Patients (%)
Duration of imatinib therapy before reduction dose, years, mean ± SD	6.95 ± 3.44
**Reasons for reducing imatinib doses** **Toxicity**	42 (80.7)
Myelosuppression	13 (25.0)
Diarrhea	5 (9.6)
Nausea and vomiting	6 (11.5)
Edema	5 (9.6)
Bone pain	3 (5.8)
Headache	2 (3.8)
Rash	8 (15.4)
**Preparation before discontinuation**	4 (7.7)
**Economic reasons**	6 (11.5)
**Clinical response before reduction dose**	
MR4	8 (15.4)
MR4.5 (or better)	44 (84.6)
**Current dose**	
200 mg/day	39 (75.0)
300 mg/day	13 (25.0)
**Current status**	
Loss of MMR	2 (3.8)
Loss of DMR^a^	5 (9.6)

^a^Five patients lost their DMR status, and two lost their MMR status.

MR, molecular response; MMR, major molecular response; DMR, deep molecular response.

### Imatinib discontinuous therapy

The results of imatinib discontinuous therapy are shown in **Table 5**. A total of 28 (11.8%) patients received imatinib discontinuous therapy. The median time to maintain DMR before discontinuation was 8.43 years (IQR: 6.88–10.45). Of the 28 patients, toxicity prompted elective discontinuation in 15 patients, and four patients desired to get pregnant. Of the 28 patients, six (2.5%) had molecular recurrence, and three (1.3%) lost the DMR. The median time to loss of MR4 and MMR were 6.45 months (IQR: 4.93–16.75) and 9.17 months (IQR: 6.87–36.98), respectively. The median TFR duration for the 15 patients who resumed TKI therapy was 15.33 months (IQR: 6.45–47.58). Four patients received a dose of 400 mg/day, 10 received 200 mg/day, and two switched to second-generation TKIs. Reasons for restarting therapy were loss of DMR (N=3), molecular recurrence (N=6), after pregnancy (N=4), and withdrawal syndrome (N=2). All patients regained MMR after further treatment for 3.38 months (IQR: 1.11–6.31) and achieved DMR with a median time of 8.27 months (IQR: 4.99–16.34). Thirteen patients (5.5%) remained in TFR for a median of 43.33 months (IQR: 14.13–61.05). No patient transformed to AP or BP or died during imatinib discontinuation treatment.

**Table 5:** Imatinib discontinuous therapy.

Elective discontinuation in sustained DMR, N=28 (11.8%)	
Duration of DMR before discontinuation, year, median (IQR)	8.43 (6.88–10.45)
Duration of TFR, month, median (IQR)	15.33 (6.45–47.58)
**Reasons for imatinib discontinuation**	
Purely elective treatment discontinuation	9 (32.1)
Elective discontinuation prompted by toxicity	15 (53.6)
Elective discontinuation to pursue pregnancy	4 (14.3)
**Resumed TKIs treatment**	15 (53.6)
Imatinib	13 (46.4)
Dasatinib	1 (3.6)
Nilotinib	1 (3.6)
**Resumed imatinib dosage**	
200 mg daily	10 (35.7)
400 mg daily	3 (10.7)
**Reasons for resumed treatment**	
TFR<12 months^a^	8 (28.6)
Loss MMR	4 (14.3)
TFR >12 months	7 (25.0)
Loss MMR	2 (7.1)
Loss DMR	3 (10.7)
Withdrawal syndrome	2 (7.1)
Time to regain MMR, month, median (IQR)	3.38 (1.11–6.31)
Time to regain DMR, month, median (IQR)	8.27 (4.99–16.34)

^a^Fifteen patients resumed TKI treatment, including four who resumed treatment after giving birth.

IQR, inter-quartile range; TKI, tyrosine kinase inhibitor; MR, molecular response; MMR, major molecular response; DMR, deep molecular response; TFR, treatment-free remission.

### Toxicity

A total of 237 patients experienced 795 treatment-emergent AEs, including 110 grade 3/4 AEs, regardless of causality. The AEs related to imatinib are shown in **Table 6**. No new, late toxicity was observed, and most AEs occurred during the first year and declined over time. Hematologic toxicities [anemia (54.0%), leukopenia (32.5%), thrombocytopenia (28.3%), and neutropenia (21.9%)], periorbital and limb edema (34.2%), diarrhea (19.8%), and rash (19.0%) were the most common AEs. In addition, the most frequent grade 3/4 AEs were neutropenia (9.3%), anemia (7.6%), thrombocytopenia (6.3%), and rash (4.2%).

**Table 6:** Adverse events of imatinib in patients with CML.

Adverse reactions	Any Grade		Grade 3/4	
	N	%	N	%
Leukopenia	77	32.5	9	3.8
Neutropenia	52	21.9	22	9.3
Anemia	128	54.0	18	7.6
Thrombocytopenia	67	28.3	15	6.3
Diarrhea	47	19.8	5	2.1
Nausea and vomiting	21	8.9	3	1.3
gastrointestinal discomfort	17	7.2	2	0.8
Musculoskeletal pain	35	14.8	8	3.4
Periorbital and limb edema	81	34.2	9	3.8
Rash	45	19.0	10	4.2
Headache	26	11.0	2	0.8
Fatigue	19	8.0	2	0.8
Conjunctival hemorrhage	5	2.1	0	0
Hyperglycemia	20	8.4	0	0
Dyslipidemia	47	19.8	2	0.8
Hyperuricemia	40	16.9	1	0.4
Hepatobiliary dysfunction	43	18.1	2	0.8
Renal insufficiency	13	5.5	0	0
Skin whitening	12	5.1	0	0

## Discussion

This study reported the long-term follow-up data of imatinib as the frontline setting in Chinese CML-CP patients. Our results highlight the safety and efficacy of imatinib therapy, and the feasibility of dose reduction and imatinib discontinuation therapy.

After more than 10 years of follow-up, the IRIS and CML study IV trials confirmed the long-term efficacy of imatinib. In the IRIS trial (6), the efficacy and tolerance of imatinib 400 mg daily were superior to interferon and cytarabine. During 18 months of follow-up, the CCyR rate of imatinib was significantly higher than that of the control group (76% *vs* 15%; P<0.001), and the 5-year cumulative CCyR rate was 87%. In addition, the OS rate was 83.3% after a median follow-up of 10.9 years. About 6.9% and 15.9% of patients stopped imatinib treatment due to unfavorable therapeutic effect or AEs, respectively. In the CML Study IV (7), newly diagnosed CML patients were treated with imatinib 800 mg daily, 400 mg daily, or 400 mg daily plus either interferon or cytarabine. During a median follow-up of 7.1 years, 64% of patients remained on imatinib therapy, and 22% switched to second-generation TKIs. At 10 years, the cumulative OS rate was 84%, and 89% and 72% achieved MMR and MR4.5. In our study, the cumulative CCyR, MMR, and MR4.5 rates were 82.6%, 80.4%, and 69.3%, respectively. In addition, 18.1% and 8.4% of patients stopped imatinib treatment owing to unsatisfactory therapeutic effect or AEs.

With the accumulated experience in using imatinib to treat CML, the efficacy of imatinib improved over time. The clinical response rates of our patients are similar to those of clinical trials, probably because we emphasized the importance of standardized management for CML patients in clinical practice. For example, after identifying the poor compliance with imatinib administration (8, 9), we equipped special clinical pharmacists to provide more specific drug education and consultation for patients to ensure they take their medication correctly. Furthermore, another important factor affecting efficacy is affordability, especially with life-long treatment. Higher out-of-pocket expenses were associated with lower quality of life and compliance (10, 11), while TKI compliance below 90% was related to a lower MMR rate (12). With the increasing availability of TKI, more CML patients are likely to receive domestic imatinib with the economic considerations in China. Moreover, all domestic imatinib have passed the consistency evaluation. This greatly reduces the economic burden on patients, improves compliance with long-term medication, and increases the therapeutic effect.

Recently, dose reduction has been used as an important attempt to ameliorate AEs and improve patients’ compliance in clinical practice (13). In the DESTINY trial (14), only 2.5% (MR4) and 18.8% (MMR) of patients lost their MMR during the first year of TKI dose reduction (50% reduction). Claudiani et al. (15) conducted a retrospective study of 246 CML patients who received low-dose TKIs treatment. The findings suggested that dose reduction is not recommended as a routine clinical practice but could be an acceptable and safe option for patients who cannot tolerate the standard-dose regimen. Cervantes et al. (16) indicated that imatinib 300 mg daily could minimize toxicity while maintaining the clinical response. Furthermore, a recent study indicated that low-dose TKIs could maintain molecular response without impairing the achievement of TFR (17). The results of these clinical trials and real-life settings suggest that TKI dose reduction is feasible in CML patients with optimal responses. In this study, 21.8% of patients received low-dose imatinib therapy, and only two patients lost their MMR status, while three lost their DMR status. Our results suggested that reducing the dosage of imatinib to 200 mg or 300 mg daily was feasible and safe in CML patients with sustained optimal response. Furthermore, the AEs related with imatinib could be dramatically improved after dose reduction.

With the development of diagnosis and treatment for CML, TFR has become a standardized management goal for CML patients (5). Imatinib treatment also enabled some patients with sustained DMR to stop treatment successfully (for >5 years), promoting TFR as a treatment goal (18, 19). The STIM (20–22) and TWISTER (23) trials suggested a safe and effective TFR. Approximately 39%–45% of patients with durable DMR who received imatinib therapy can remain TFR for three years or longer (24). After six years of imatinib treatment, the eligibility rate of TFR attempt was estimated to be 21.6% (25). Therefore, the total percentage of patients who received imatinib therapy with stable TFR was approximately 10%. In this study, 11.8% of patients attempted imatinib discontinuation after a median duration of DMR of 8.43 years, and 5.5% were still on TFR. Notably, some patients are more inclined to reduce TKI doses than full discontinuation in real-life settings, primarily because they are concerned about the recurrence of full discontinuation. Full discontinuation of TKI therapy requires more molecular monitoring at the early period of the discontinuation, which patients are reluctant to do. In addition, with the development of medical insurance and generic TKIs, the main problem related to AEs can be significantly improved by dose reduction.

Eight-year safety data from the CML Study IV (26) indicated that 76% of patients treated with imatinib had AEs, most AEs were mild and manageable, and only 22% were grade 3/4. In this study, most AEs were manageable and may occasionally lead to dose reduction or discontinuation of imatinib. No new, late toxicity was observed, and most AEs often occurred during the first year and declined over time. The most frequent grade 3/4 AEs were neutropenia (9.3%), anemia (7.6%), thrombocytopenia (6.3%), and rash (4.2%).

The individual characteristics of CML patients, TKIs compliance, lifestyle preferences, comorbidities, toxicity profiles of TKIs, and physician-clinical center experience are among the critical factors to consider while deciding on the proper first-line TKI in newly diagnosed CML patients (27). Imatinib is still a cost-effective option for first-line treatment of some CML patients in China, especially for elderly patients. In conclusion, this study confirmed the long-term efficacy and safety of imatinib treatment for Chinese CML patients and demonstrated the feasibility of dose reduction and TFR attempts in patients who received imatinib in real-life settings.

This study had some limitations. Firstly, it was a retrospective and observational study, which might be subject to selection bias. In addition, some patients have been diagnosed for a long time and lost their data, making it impossible to calculate Sokal or ELTS risk score at diagnosis. A total of 87 (36.7%) of the 237 CML patients provided data on their Sokal scores at diagnosis. Among the 87 patients, 51 (58.6%) had low-risk, 28 (32.2%) had intermediate-risk, and 8 (9.2%) had high-risk. Moreover, some patients were lost to follow-up, so we could not evaluate the OS rate in this study. Nevertheless, we will continue to collect the long-term outcomes of patients. Finally, clinical response to imatinib treatment could not be analyzed in all patients owing to poor financial condition and compliance.

## Data availability statement

The original contributions presented in the study are included in the article/supplementary material. Further inquiries can be directed to the corresponding author.

## Ethics statement

This study has been approved by the institutional ethics committee of Tongji Medical College, Huazhong University of Science and Technology (Wuhan, China) ([2021] 0784). The patients/participants provided their written informed consent to participate in this study.

## Author contributions

FC: Writing - Original Draft, Data Curation, Data Analysis. GY: Data Analysis, Writing - Original Draft. QL: Data Curation, Writing - Original Draft. ZC: Data Curation. WL: Conceptualization, Visualization, Revised Draft. All authors contributed to the article and approved the submitted version.
